# Hospital Administration.—Alcohol at Provincial Hospitals

**Published:** 1887-09-24

**Authors:** 


### Hospital Administration.—Alcohol at Provincial Hospitals.

The following Table shows that an alteration in the accounts of these Institutions in the direction of fuller information is needed, so that accurate comparisons may be possible. So far as the facts are ascertainable, it will be seen on reference to the article last week (*p.* 413), and to previous Tables (*pp.* 5, 41, 75, *Vol. 1*), that the position of the alcohol question is pretty much the same in Metropolitan and Provincial Hospitals.

*Value of the Consumption of Milk and Alcohol at Twenty-one Provincial Hospitals, during the years 1875 and 1885 respectively.*

| Name of Hospital. | 1875 Total Consumption of Milk. | 1875 Wine and Spirits. | 1875 Malt Liquors: Staff and Patients. | 1875 Spirits of Wine. | 1875 Total Consumption of Alcohol. | 1875 No. of In-Patients | 1885. Total Consumption of Milk. | 1885. Wine and Spirits. | 1885. Malt Liquors: Staff and Patients. | 1885. Spirits of Wine. | 1885. Total Consumption of Alcohol. | 1885. No. of In-Patients | 1875 and 1885 compared. Percentage of Increase (+) or Decrease (—): In Number of In-Patients | in consumption of Milk. | in consumption of Alcohol |
|---|---|---|---|---|---|---|---|---|---|---|---|---|---|---|---|
| | £ s. d. | £ s. d. | £ s. d. | £ s. d. | £ s. d. | | £ s. d. | £ s. d. | £ s. d. | £ s. d. | £ s. d. | | | | |
| Birkenhead Borough Hospital | 81 6 2 | 99 14 3 | 61 16 0 | — | 161 10 3 | — | 135 14 5 | 27 0 0 | 47 15 9 | — | 74 15 9 | — | — | +66·7 | —53·7 |
| Bristol General Hospital | 341 0 9 | 98 16 0 | 129 14 2 | 27 16 10 | 256 7 0 | 1,555 | 562 8 6 | 190 13 3 | 97 4 2 | 39 10 0 | 327 7 5 | 2,167 | +39·3 | +64·8 | +27·7 |
| Bristol Royal Infirmary | 442 0 0 | 423 0 0 | 333 0 0 | no record. | 756 0 0 | 2,192 | 610 0 0 | 208 0 0 | 213 0 0 | 15 0 0 | 436 0 0 | 3,434 | +56·66 | +38·0 | —42·3 |
| Chester General Infirmary | 171 0 0 | 29 0 0 | 59 0 0 | 41 0 0 | 129 0 0 | 794 | 203 0 0 | 62 0 0 | ‡5 13 0 | 60 0 0 | 127 13 0 | 907 | +14·2 | +18·7 | —1·0 |
| Derbyshire General Infirmary | 211 0 0 | 96 0 0 | 183 0 0 | — | 279 0 0 | 1,178 | 294 0 0 | 89 0 0 | 104 0 0 | — | 193 0 0 | 1,401 | +15.77 | +39·3 | —30·8 |
| Devon and Exeter Hospital | 250 0 0 | 201 0 0 | 363 0 0 | 25 0 0 | 589 0 0 | 1,217 | 430 0 0 | 224 0 0 | 158 0 0 | 25 0 0 | 407 0 0 | 1,103 | —9·4 | +7·2 | —30·9 |
| Kent and Canterbury Hospital | 106 17 11 | 98 5 0 | 142 0 6 | — | 240 5 6 | 599 | 263 6 7 | 65 11 0 | 67 8 0 | — | 132 19 0 | 706 | +17·9 | +143·9 | —44·6 |
| Leicester Infirmary and Fever Hospital | 456 0 0 | 343 0 0 | 293 0 0 | 47 0 0 | 683 0 0 | 2,077 | 563 0 0 | 156 0 0 | 72 0 0 | 6 0 0 | 234 0 0 | 2,531 | +21·9 | +23·5 | —65·7 |
| Lincoln County Hospital | 264 0 0 | 171 0 0 | 129 0 0 | — | 300 0 0 | 667 | 330 0 0 | 118 0 0 | 49 0 0 | — | 167 0 0 | 720 | +7·9 | +25·0 | —44·3 |
| Liverpool Royal Southern Hospital | 400 0 0 | 135 13 10 | 220 16 0 | — | 356 9 10 | 2,005 | 541 0 0 | 118 7 4 | 91 8 6 | — | 209 15 10 | 1,725 | +13·9 | +35·2 | —41·3 |
| Manchester Royal Infirmary | 485 13 3 | 751 8 3 | 424 15 0 | 256 0 0 | 1,432 3 3 | 3,125 | 990 17 5 | 52 6 10 | 148 9 11 | 160 6 1 | 361 2 10 | 4,455 | +42·6 | +103·9 | —74·8 |
| Newcastle-upon-Tyne Infirmary | 736 3 8 | 208 6 9 | 201 6 10 | — | 409 13 7 | 1,679 | 1,105 11 6 | 230 8 0 | 90 18 3 | — | 321 6 3 | 2,846 | +6·9 | +50·1 | —21·5 |
| Northampton General Infirmary | 100 0 0 | 251 0 0 | 323 0 0 | 52 0 0 | 626 0 0 | — | 218 0 0 | 171 0 0 | 135 0 0 | 49 0 0 | 355 0 0 | 1,702 | — | +118·0 | —44·3 |
| North Staffordshire Infirmary | 216 0 0 | 99 0 0 | 238 0 0 | — | 337 0 0 | 1,270 | 412 0 0 | 59 0 0 | 127 0 0 | — | 186 0 0 | 1,660 | +30·5 | +90·7 | —44·8 |
| Nottingham General Hospital | 338 2 7 | 82 2 6 | 238 12 0 | — | 320 14 6 | 1,267 | 494 8 2 | 60 16 1 | 103 7 6 | — | 164 3 7 | 1,602 | +26·4 | +46·1 | —48·9 |
| Oldham Infirmary | 91 0 0 | 52 0 0 | 38 0 0 | — | 90 0 0 | 320 | 215 0 0 | 19 0 0 | ‡19 0 0 | — | 38 0 0 | 593 | +85·3 | +136·3 | —57·8 |
| Radcliffe (Oxford) Infirmary | 172 14 0 | 177 17 6 | 238 7 3 | 60 4 5 | 476 9 2 | — | 375 1 4 | 86 11 0 | 134 18 0 | 27 7 7 | 248 16 7 | 1,461 | — | +116·8 | —47·9 |
| Royal Hants County Hospital | 225 5 11 | 101 13 1 | 101 13 0 | 18 6 6 | 221 12 7 | 738 | 177 3 0 | 33 9 3 | 34 19 9 | 21 3 3 | 89 12 3 | 578 | —21·7 | —21·3 | —59·5 |
| Royal South Hants Infirmary | 92 12 5 | 94 18 0 | 89 9 8 | — | 184 7 8 | 769 | 213 7 2 | 38 3 6 | ‡17 16 8 | — | 56 0 2 | 1,018 | +32·4 | +130·2 | —69·6 |
| Salop Infirmary.. .. | 181 19 9 | 202 9 7 | 342 12 7 | 62 14 0 | 607 16 2 | 835 | 255 14 1 | 86 17 10 | 93 5 6 | 42 12 9 | 222 16 1 | 1,056 | +26·4 | +40·7 | —63·3 |
| Sunderland and Bishopwearmouth Infirmary | 202 1 6 | 123 2 6 | — | — | 123 2 6 | 598 | 407 8 6 | 16 6 1 | ‡7 7 6 | — | 23 13 7 | 1,438 | +140·5 | +101·5 | —81·0 |
| Total.. .. .. | 5,564 17 11 | 3,839 7 3 | 4,150 3 0 | 590 1 9 | 8,579 12 0 | — | 8,797 0 8 | 2,112 10 2 | 1,817 12 6 | 445 19 8 | 4,376 2 4 | — | — | +58·1 | —49·0 |

‡ Patients only.

